# Transmembrane Protein 100 Inhibits the Progression of Colorectal Cancer by Promoting the Ubiquitin/Proteasome Degradation of HIF-1α

**DOI:** 10.3389/fonc.2022.899385

**Published:** 2022-07-19

**Authors:** Ying Zheng, Yitong Zhao, Jiong Jiang, Baicang Zou, Lei Dong

**Affiliations:** ^1^ Department of Digestive Disease and Gastrointestinal Motility Research Room, The second Affiliated Hospital of Xi’an Jiaotong University, Xi’an, China; ^2^ School of Basic Medical Sciences, Xi’an Jiaotong University Health Science Center, Xi’an, China

**Keywords:** TMEM100, HIF-1α, ubiquitination/proteasome pathway, angiogenesis, colorectal cancer

## Abstract

Transmembrane protein 100 (TMEM100) is involved in embryonic cardiovascular system development. However, the biological role of TMEM100 in human cancers, particularly colorectal cancer (CRC), is unclear. In this study, tissue microarrays were stained using immunohistochemistry methods to evaluate the association between TMEM100 levels and clinic-pathological features for CRC. Kaplan–Meier and log-rank tests revealed that decreased levels of TMEM100 correlated with shorter overall survival. Cox regression revealed that reduced levels of TMEM100 was an independent prognostic factor for detrimental survival in CRC. A lentiviral vector was used to overexpress TMEM100 in HCT116 cells, and small interfering RNA was used to knockdown TMEM100 in SW480 cells. The CCK-8 assay, colony formation analysis, cell cycle analysis, cell migration assay, mouse xenograft model and mouse lung metastasis model showed that TMEM100 suppressed CRC cell proliferation and migration *in vitro* and *in vivo*. IHC scores of TMEM100 and HIF-1α were significantly negatively correlated. A half-time determination analysis in which cells were treated with cycloheximide revealed that TMEM100 shortened the HIF-1α half-life. Further immunoprecipitation experimental results showed that TMEM100 promoted the ubiquitination of HIF-1α, which caused HIF-1α degradation *via* the 26S proteasome pathway. Angiogenesis assay and migration assay results revealed that TMEM100 suppressed the migration and angiogenesis induction capacities of HCT116 cells, but this inhibitory effect was abolished when HIF-1α degradation was blocked by MG132 treatment. These results indicated that TMEM100 inhibited the migration and the angiogenesis induction capacities of CRC cells by enhancing HIF-1α degradation *via* ubiquitination/proteasome pathway.

## Introduction

The mortality of colorectal cancer (CRC) is third highest among all tumors, causing a significant financial burden to society (1, 2). According to a global statistical survey, in 2018, more than 1.8 million new CRC diagnoses were recorded, which accounts for approximately 10% of the overall number of new cancer cases worldwide (2). Approximately 30% of CRC patients have progressed to advanced or accompanying distant organ metastasis when diagnosed with CRC, and approximately 86% of patients with advanced cancer die within 5 years of diagnosis. Considering the above situation, improvement of diagnostic approaches and development of novel treatment strategies for CRC patients are extremely urgent matters.

During the development of the embryo, transmembrane protein 100 (TMEM100) is expressed in arterial endothelial cells. Targeted dysregulation of TMEM100 causes embryonic lethality with severe vascular formation abnormalities (3, 4). TMEM100 is involved in the development of embryonic cardiovascular system which includes endothelial cell migration, proliferation, neovascular reorganization, stabilization of endothelial cells and establishment of vascular architecture (3). This suggests that TMEM100 is most likely to be involved in processes such as malignant tumor metastasis and the induction of microangiogenesis. TMEM100 exhibited a clear inhibition of metastasis and proliferation in lung cancer, prostate cancer (PC), hepatocellular carcinoma (HCC) and gastric cancer (GC), and is correlated with the prognostic outcomes of above malignancies (5–11). In lung adenocarcinoma, miR-421 could be sponged by circ_0000567 and directly target TMEM100 mRNA, then regulated the migration and invasion (5). And Histone deacetylase 6 (HDAC6) acted as a metastasis supporter induced the Wnt/β-catenin signaling pathway by suppressing TMEM100 expression in non-small cell lung cancer (NSCLC) (6). In PC, GATA binding protein 5-mediated transcriptional activation of TMEM100 suppresses cell proliferation, migration and epithelial-to-mesenchymal transition (7). Elevated TMEM100 level inhibits pulmonary metastasis of GC cells and enhances xenograft tumor sensitivity to 5-FU therapy (9). A study at the cellular level revealed that TMEM100 modulates TGF-β signaling pathway to inhibit CRC progression (12). However, the significance of TMEM100 expression in CRC tissues remains unclear.

Hypoxia-inducible factor-1α (HIF-1α), a key protein in cellular response to the hypoxia, has clear tumorigenesis effects (13–15). Angiogenesis induced by the HIF-1α/VEGF pathway is pivotal for tumor migration and invasion in CRC (16, 17). In consideration of the role of TMEM100 on angiogenesis, endothelial cell migration and proliferation during embryonic development, we speculate that TMEM100 was potentially associated with HIF-1α in CRC. However, the relationship among TMEM100, HIF-1α and CRC has never been reported.

## Materials and Methods

### Clinical Samples and Tissue Microarray (TMA)

Twenty-three primary paired CRC samples containing adjacent tissues from the Second Affiliated Hospital of Xi’an Jiaotong University (Xi’an, China) were subjected to western blotting as well as RT–qPCR. Prior to sample collection, each patient signed an informed consent form. Permission for this study was obtained from the Clinical Research Ethics Committee of the Second Affiliated Hospital of Xi’an Jiaotong University (#2019022).

The TMAs (HColA180Su15, HCol-Ade180CS-01) with 164 adjacent and 186 CRC tissues were procured from Shanghai Outdo Biotech (China). The CRC tissues on the TMAs correspond to the clinicopathological parameters (age, gender, tumor diameter, tumor location, tumor invasion, lymph node metastasis, organ metastasis and AJCC stage) of the CRC patients. And 101 CRC tissues correspond to the follow-up information of CRC patients. Operative period was from January 2009 to October 2009, while the follow-up time was July 2015. Follow-up intervals were 6.7 to 7.2 years.

### Cell Lines and Culture

HT29, CaCo2, HCT116, SW480, DLD1 and HUVEC cell lines were acquired from the American Type Culture Collection (ATCC). NCM460 and HCoEpic normal human colon cell lines and Lovo cell line were acquired from the Cell Bank of the Chinese Academy of Sciences (Shanghai, China). PCR tests did not reveal any mycoplasma contamination. DLD1 and Lovo cells were grown in RPMI 1640 medium, and other cells were grown in high-glucose DMEM with 100 mL/L fetal bovine serum (FBS, Gemini, USA) in a humidified 5% CO_2_ incubator at 37°C. To induce hypoxia, cells were cultured with 1% O_2_, 94% N_2_ and 5% CO_2_ (vol/vol) at 37°C.

### Lentiviral Infections and Generation of a Stable Cell Line

HCT116 cells were infected with the lentiviral vectors for TMEM100 overexpression or negative control lentiviral vectors (Shanghai Genechem Co., Ltd.). The efficiency of infection was determined by western blotting. Then, we used a medium with 2 μg/mL puromycin to establish stable overexpressing (LV-TMEM100) or negative control (LV-vector) HCT116 cell lines.

### SiRNA Transfection

SW480 cells were transfected with TMEM100 specific siRNAs (GenePharma, Shanghai, China). The siRNA sequences were: si-TMEM100-1: 5`-CAGACUUUAUGUUCAUAGUUCUUCCUC-3`; si-TMEM100-2: 5`-CUUCCACAACUACAUAGGGUAUUGUUU-3`; the negative control sequence (si-NC) was UUC 5`-UCCGAACGUGUCACGUTT-3`. The lipofectamine 2000 reagent (Invitrogen, Carlsbad, CA) was supplemented to enable transfection, as instructed by the manufacturer. siRNAs were transfected for 6 h at a final concentration of 50 nM.

### Immunohistochemistry (IHC) and Evaluation of Immunostaining Intensity

Paraffin-embedded sections and the TMAs were dewaxed with dimethylbenzene and gradient alcohol (100%, 95%, 85% and 75%). The antigen retrieval was done by microwaving sections in 0.01 M sodium citrate, pH 6.0. Then, incubation of slides was done for 20 min in the presence of 3% hydrogen peroxide after which they were incubated in the presence of goat serum at room temperature (RT) for 30 min. Then, overnight incubation of slides was done in the presence of primary antibodies (anti-TMEM100 (GeneTex, GTX83507); anti-Ki-67 (Servicebio, GB111499); anti-HIF-1α (Servicebio, GB13031-1); anti-CD34 (Servicebio, GB13013)) at 4°C followed by supplementation with biotinylated anti-IgG and incubation at 25°C for 1 h. After incubation for 30 min with streptomycin-HRP, sections were DAB-stained, counterstained with hematoxylin, washed with water, dehydrated by an alcohol gradient (80%, 90% and 100%) and dimethylbenzene. Lastly, neutral balsam and coverslips were used to seal the slides.

For the immunostaining intensity evaluation of TMAs, two pathologists that were not aware of clinicopathological features as well as patient outcomes independently scored the tissue microarrays. Immunoreactivity was grouped into 5 grades (% score) based on stained cells % as: 0, no staining; 1, 1-25%; 2, 26-50%; 3, 51-75%; and 4, >75%. Staining intensities were assigned into 4 grades (intensity score) as follows: 0, negative; 1, weak; 2, moderate; and 3, strong. Then, a final overall histological score was calculated by multiplication of the two values (18). An overall score of 0–12 was calculated and graded as low (score ≤ 5) or high (score>5) in order to estimate the relationship between the expression of TMEM100 and the clinicopathological parameters of CRC patients.

For the immunostaining intensity evaluation of murine subcutaneous tumors’ tissues, Image-Pro Plus 6.0 software (Media Cybernetics, Maryland United States) was used to calculate the ratio of the positively stained area to the area of the field of view, and the integral optical density from the five fields was taken.

### Hematoxylin-Eosin (HE) Staining

Paraffin-embedded sections were dewaxed with xylene, dehydrated with gradient ethanol, stained with Harris’s hematoxylin for 5 min, differentiated with 0.1% hydrochloric acid alcohol, stained with 1% eosin for 2 min, dehydrated by gradient ethanol, cleared in xylene, mounted with neutral gum, and observed and photographed under a microscope.

### Western Blotting

Total proteins from cultured cells and clinical patient samples were extracted by the RIPA buffer (Beyotime, China) containing a protease suppressor cocktail (1:100, Bimake). For the western blotting assay, protein separation was achieved using 10% sodium dodecyl sulfate–polyacrylamide gel electrophoresis (SDS–PAGE) after which they were transferred to polyvinylidene difluoride membranes. Membranes were blocked with Tris-buffered saline supplemented with 0.1 mL/L Tween 20 (TBST) and fresh prepared 100 g/L non-fat milk for 2 h at 25°C, followed by incubation at 4°C overnight in the presence of various primary antibodies (anti-TMEM100 (GeneTex, GTX83507); anti-CDK2 (Abcam, ab32147); anti-Cyclin D1 (Abcam, ab40754); anti-PCNA (Abcam, ab92552); anti-Bcl-2 (Abcam, ab182858); anti-caspase3 (Abcam, ab32351); anti-cleaved caspase3 (Abcam, ab32042); anti-PARP1 (Abcam, ab191217); anti-cleaved PARP1 (Abcam, ab32064); anti-E-cadherin (Abcam, ab40772); anti-Vimentin (Abcam, ab8978); anti-HIF-1α (Abcam, ab1) and anti-β-actin (Fdbio science, AP0060)). Then, they were washed using a TBST buffer followed by 1 h of incubation in the presence of goat anti-rabbit IgG-HRP antibodies (1:10000, Zhuangzhi, EK020) and goat anti-mouse IgG-HRP antibody (1:10000, Fdbio science, FDR007) at 25°C. Visualization of immunoreactivity was done using an ECL substrate (Bio–Rad, Hercules, CA) on the Gene Gnome XRQ System. ImageJ (National Institute of Health, MD) was used for densitometric assessments.

### Immunoprecipitation (IP) Assay

HCT116 cells were infected with LV-TMEM100 or LV-vector lentiviruses, and then cultured for 48 h under hypoxic conditions. Then total proteins from cultured cells were extracted by the RIPA buffer (Beyotime, China) containing a protease suppressor cocktail (1:100, Bimake). For the IP assay, first, anti-HIF-1α was mixed with Protein A/G PLUS-agarose (RuiSike Science & Technology Co., Ltd, China) at 4°C for 12 h. Then, antibody- agarose complex was incubated with the protein samples for 12 h at 4°C. After washing using a washing buffer, the antibody-agarose-protein complex was obtained and mixed with SDS-PAGE sample loading buffer (Beyotime, China). Then the mixture was heated in a Dry thermostat (Beyotime, China) at 95°C for 10 min. The protein solutions were further evaluated by the western blotting assay.

### Real-Time Quantitative PCR (RT–qPCR) Assay

The TRIzol reagent (Invitrogen, USA) was used for total RNA extraction, as denoted by the manufacturer. Then, the Transcript First-Strand cDNA Synthesis Kit (Roche, Denmark) was used to reverse-transcribe 2 µg of total RNA to cDNA. RT–qPCR was done using a FastStart Universal SYBR Green Master Mix (Roche, Denmark) on a 7500 Fast Real-Time PCR System (Applied Biosystems, Foster City, CA, USA). Relative levels of target genes were evaluated by the 2^-△△Ct^ approach, with normalization to β-actin. Primer sequences for this assay were: β-actin (forward 5’-GGCACCACACCTTCTACAATGAGC-3’, reverse 5’-GATAGCACAGCCTGGATAGCAACG-3’), TMEM100 (forward 5’-GGAGAAGAGCCCCAAGAGTG -3’, reverse 5’- TGCAGCGGTAGCAGGAGA-3’), HIF-1α (forward: 5’-AGCTTCTGTTATGAGGCTCACC-3’, reverse: 5’-TGACTTGATGTTCATCGTCCTC-3’).

### Cell Viability Analysis

Cell viabilities were assessed by the Cell Counting Kit-8 (CCK-8, 7sea Pharmatech Co., Ltd). Cells were seeded in each well of a 96-well plate in 200 μL of medium (5 × 10^3^ cells/well) and then the CCK-8 reagent (10 μL) dissolved in 90 μL of serum-free medium was added to each well 24 h, 48 h, 72 h, and 96 h after plating. After the addition of the CCK-8 solution, cell incubation was done at 37°C for 30 min. Absorbance was measured at 450 nm.

### Colony Formation Assay

Cells were cultured into 6-well plates (1×10^3^ cells/well) followed by incubation for 14 days at 37°C. Then, they were fixed in paraformaldehyde (4%) followed by crystal violet staining. Colony counts were done visually.

### Cell Migration Assay

Cell migration assay was assessed by using a Transwell chamber with a pore size of 8 μm (Corning, USA). A total of 8×10^4^ cells were seeded into the upper chamber in 200 µL of serum-free DMEM. Then, 600 µL of DMEM with 200 mL/L FBS was added to the lower chamber. After incubation at 37°C with 50 mL/L CO_2_ for 24 h, the cells that had migrated through the membrane were fixed in 40 mL/L paraformaldehyde and stained with crystal violet, while the cells in the upper chamber were carefully removed using a cotton swab. After drying, the cells that had migrated were counted with a microscope at 100× magnification in 5 random fields.

### Cell Invasion Assay

Cell invasion assay was assessed by using a Transwell chamber with a pore size of 8 μm (Corning, USA). For cell invasion assay, the upper chambers were covered with Matrigel (60 μL, 200 mg/mL, BD Biosciences, San Diego, CA, USA), and concreted for 4 h in the incubator. The next steps were same as the cell migration assay.

### Flow Cytometry

For cell cycle assessment, 2×10^5^ cells in each group were grown in 6-well plates in serum-free media for 36 h to maintain the same cell cycle. Then the media was replaced by high-glucose DMEM with 100 mL/L fetal bovine serum to culture cells for another 24 h. Adherent cells were digested into a centrifuge tube and washed gently to remove trypsin. Then 70% ice ethanol was added to fix the cells overnight at 4 °C. The next day, fixed cells were washed and resuspended in 500 μL PI/RNase Staining Buffer (BD Biosciences, Franklin Lakes, NJ, USA). After 15 min of incubation in the dark at 25°C, flow cytometry (BD Biosciences, Franklin Lakes, NJ, USA) was performed to assess cell cycle phases.

For cell apoptosis analysis, a PE Annexin V/7-amino-actinomycin Detection (7-AAD) Kit (BD Biosciences) was used according to the manufacturer’s instructions. To determine the apoptosis fraction, cells without PE Annexin V or 7-AAD staining were recognized as negative control. And cells staining with only PE Annexin V were in early apoptosis stage. Cells staining with PE Annexin V and 7-AAD were in late apoptosis stage.

### Enzyme-Linked Immunosorbent Assay (ELISA)

The amount of cell-secreted VEGF proteins in the medium were evaluated by a VEGF ELISA kit, as instructed by the manufacturer (#KE00085, Proteintech, CA, USA).

### *In Vitro* Tube Formation Assay

This assay consisted of three groups: the control group (LV-vector group), the TMEM100 overexpression group (LV-TMEM100 group), and the TMEM100 overexpression with MG132 treatment group (LV-TMEM100 + MG132 group). HCT116 cells (20×10^4^) infected with LV-vector virus were inoculated in 6-well cell culture plates in the LV-vector group. HCT116 cells (20×10^4^) infected with LV-TMEM100 virus were inoculated into 6-well cell culture plates in the LV-TMEM100 group and the “LV-TMEM100+MG132” group. For the “LV-TMEM100 + MG132” group, MG132 was supplemented to the medium and cultured for 42 h before media collecting. The final MG132 concentration was 1 μM. All cells were grown in hypoxic environments for a total of 48 h. Then the media in the indicated groups were collected. Then HUVECs (1×10^4^ cells/well) were inoculated in 48-well cell culture plates precoated with polymerized Matrigel (BD Biosciences, San Diego, CA, USA) followed by incubation at 37°C for 4 h in conditioned media derived from the indicated cells under normoxic conditions. Changes in cellular morphology were microscopically observed. Total tube lengths in five random view-fields/well were determined by ImageJ software, and the mean value obtained. This assay was performed in triplicate.

### Mouse Models

The *in vivo* experiment was permitted by the Animal Care Committee of Xi’an Jiao Tong University (NO.XJTULAC2019-988). Athymic nude male mice (BALB/c, 4 weeks old) were acclimatized to laboratory conditions (12 h/12 h dark/light, 23°C, 50% humidity, and ad libitum provision of water and food) for 2 weeks before experimentation. The animals were maintained in specific pathogen-free environments. They were age-matched, randomized into groups (n=5/group), and randomly kept in various squirrel cages.

For xenograft tumor models, every mouse was subcutaneously administred with 7×10^6^ indicated cells into the left groin. The long diameter (R) and short diameter (r) of subcutaneous tumor were measured every five days using a vernier caliper. The tumor volume was calculated following the equation: Volume = (R×r^2^)/2. After 3 weeks, the mice were euthanized by an overdose of barbiturate (intravenous injection, 150 mg/kg pentobarbital sodium) to collect tissues. For lung metastasis models, the tail vein of each mouse was injected with 8×10^6^ of the indicated cells. After one month, mice were euthanized by an overdose of barbiturate to collect tissues. Murine tissues fixed in 10% formaldehyde were cleared in xylene, dehydrated with gradient ethanol, embedded in paraffin, and serially sectioned at 5 μm for HE staining and IHC. The numbers and sizes of metastatic tumors in the lungs from mice were evaluated by two pathologists who were blinded to mice grouping information. After which lung tissues were subjected to HE staining.

### Statistical Analysis

GraphPad Prism 8.0.1 (La Jolla, CA, USA) and SPSS 22.0 (United States) were used for data analyses. The hazard ratio and its 95% confidence interval were calculated by Cox proportional hazards regression. One-way ANOVA, chi-square test, or Fisher’s exact tests were used to assess the associations between TMEM100 levels and clinic-pathological features. The t test or the one-way ANOVA were used for analysis of continuous data. Kaplan–Meier and log-rank tests were used to estimate the overall survival rates by GraphPad Prism 8.0.1. Randomization and blinding were done in all assays. Data are shown as mean ± SD, *p*<0.05 denoted significance.

## Results

### TMEM100 Is Suppressed in Human CRC Tissues, and Was Established to be an Independent Prognostic Factor Indicating Detrimental Prognosis in CRC

**Figure 1:**
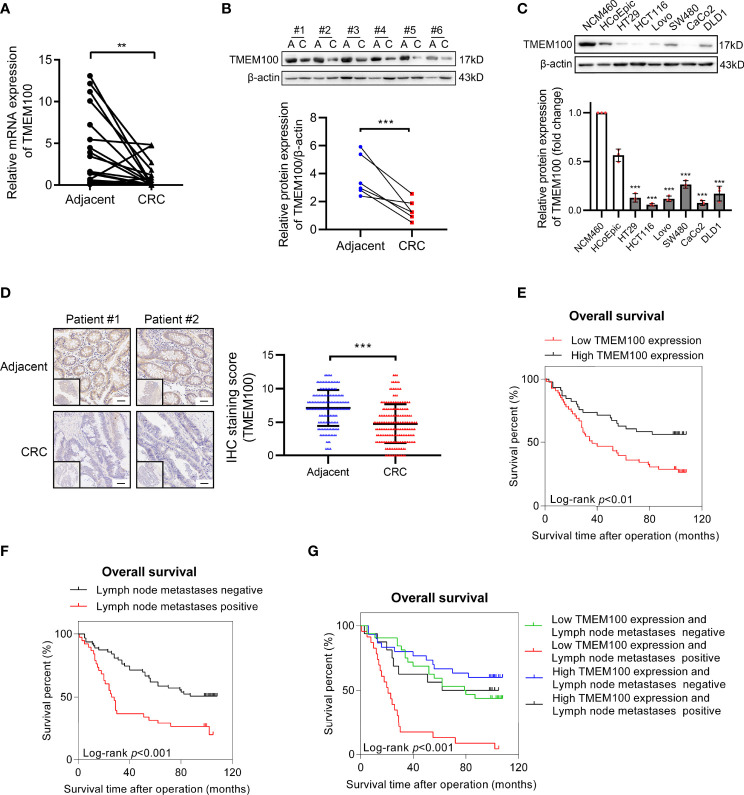
TMEM100 is downregulated in CRC tissues and reduced TMEM100 expression indicated detrimental prognosis. **(A)** Relative mRNA expression level of TMEM100 in 23 CRC tissues and paired adjacent tissues was evaluated by RT-qPCR. Two-sided t-test. **(B)** Western blotting analysis of TMEM100 in 6 paired adjacent tissues and CRC tissues. β-actin was used as a reference control. Two-sided paired t-test. **(C)** Western blotting analysis of TMEM100 level in six human CRC cell lines and two human normal intestinal epithelial cell lines (***p < 0.001 *vs* NCM460). β-actin was used as a reference control. Two-sided t-test. **(D)** Representative images of IHC staining of TMEM100 in 186 CRC tissues and 164 adjacent tissues in the tissue microarray. Scale bar = 50 μm. Two-sided t-test. **(E, F, G)** Overall survival was defined as the interval between the date of surgery and the date of death or last follow-up. The CRC patients (n=101) with lower TMEM100 level indicated detrimental prognosis **(E)**. The CRC patients (n=101) with positive regional lymph node metastasis indicated detrimental prognosis **(F)**. The CRC patients (n=101) with both lower TMEM100 level and positive regional lymph node metastasis indicated worse prognosis **(G)**. Data are presented as the mean ± SD. **p<0.01, *** *p*<0.001.

Compared to normal tissues (8.7%), mRNA levels of TMEM100 were downregulated in 21 out of 23 (91.3%) human CRC tissues (**Figure 1A**). In six pairs of CRC tissues, TMEM100 protein levels were markedly suppressed in CRC tissues, relative to normal adjacent tissues (**Figure 1B**). In comparison with that in normal colon epithelial cell line (NCM460), TMEM100 protein levels were downregulated in all 6 CRC cell lines (HT-29, HCT116, Lovo, SW480, CaCo2, and DLD1) (**Figure 1C**). Then, we further evaluated TMEM100 expression in 186 CRC and 164 adjacent tissues in tissues microarrays using IHC, and revealed that TMEM100 was dramatically suppressed in CRC tissues relative to adjacent tissues (**Figure 1D**). These findings imply that downregulation of TMEM100 plays a role in the pathogenesis of CRC.

The TMEM100 protein level was tested in 186 CRC tissues and 164 adjacent tissues in the tissue microarray using IHC. The higher and lower expression levels of TMEM100 were evaluated semiquantitatively by the staining intensity (high score: 6-12; low score: 1-5). Futher statistical analysis results showed the lower TMEM100 protein levels were associated with age, tumor diameter, tumor invasion, lymph node metastasis and metastasis (**Table 1**). The Kaplan–Meier survival analyses revealed that lower relative TMEM100 protein levels in CRC patients correlated with worse overall survival compared with higher TMEM100 expression in CRC patients (**Figure 1E**). And the CRC patients with low TMEM100 level and positive regional lymph node metastasis indicates detrimental prognosis (**Figures 1F, G**). The univariate and multivariate Cox regression results indicated that lower TMEM100 protein levels is an independent risk factor for a detrimental prognostic outcomes in CRC patients (**Tables 2**, **3**).

**Table 1:** The association of TMEM100 expression with the clinicopathological characteristics of 186 CRC patients.

Clinicopathological features	TMEM100 level		*p* value
	low (%)	high (%)	
Age (years)^1^			0.007
≤60	24 (21.2)	26 (40.0)	
>60	89 (78.8)	39 (60.0)	
Gender^2^			0.620
Male	68 (58.6)	43 (62.3)	
Female	48 (41.4)	26 (37.7)	
Tumor diameter^3^			0.042
≤5 cm	60 (51.7)	45 (67.2)	
>5 cm	56 (48.3)	22 (32.8)	
Location^4^			0.350
Colon ascendens	41 (36.3)	16 (24.2)	
Colon transversum	19 (16.8)	13 (19.7)	
Colon descendens	11 (9.7)	10 (15.2)	
Colon sigmoideum/rectum	42 (37.2)	27 (40.9)	
Tumor invasion^5^			0.006
T_1_/T_2_	8 (7.1)	14 (21.2)	
T_3_/T_4_	104 (92.9)	52 (78.8)	
Lymph node metastasis			0.001
Negative	54 (46.2)	49 (71.0)	
Positive	63 (53.8)	20 (29.0)	
Metastasis			0.033
no	103 (88.0)	67 (97.1)	
yes	14 (12.0)	2 (2.9)	
AJCC stage^6^			<0.001
I-II	51 (43.6)	48 (70.6)	
III-IV	66 (56.4)	20 (29.4)	

^1^The age in 8 patients cannot be accessed.

^2^The gender in 1 patient cannot be accessed.

^3^The tumor size of cancer in 3 patients cannot be accessed.

^4^The location in 7 patients cannot be accessed.

^5^The tumor invasion in 8 patients cannot be accessed.

^6^The AJCC stage in 1 patient cannot be accessed.

**Table 2:** Prognostic factors in 101 CRC patients by univariate Cox proportional hazards model analysis.

Parameter	Coefficient	Standard Error	HR	95% confidence	*p* value
				Interval for HR	
Age (≤60 *vs.*>60)^1^	0.506	0.348	1.658	0.838-3.280	0.146
Tumor diameter (≤5 cm *vs.* >5 cm)^2^	-0.042	0.263	0.959	0.572-1.606	0.872
Tumor invasion (T_1_/T_2_ *vs.*T_3_/T_4_)^3^	0.440	0.360	1.553	0.767-3.146	0.221
Lymph node metastasis (Negative vs. Positive)	0.922	0.261	2.514	1.507-4.196	<0.001
Metastasis (no *vs.* yes)	2.677	0.653	14.536	4.040-52.294	<0.001
AJCC stage (I-II *vs*. III-IV)^4^	0.960	0.264	2.611	1.557-4.376	<0.001
TMEM100 level (low *vs.* high)	-0.743	0.275	0.475	0.277-0.815	<0.01

^1^The age in 8 patients cannot be accessed.

^2^The tumor size of cancer in 3 patients cannot be accessed.

^3^The tumor invasion in 8 patients cannot be accessed.

^4^The AJCC stage in 1 patient cannot be accessed.

**Table 3:** Prognostic factors in 101 CRC patients by multivariate Cox proportional hazards model analysis*.

Parameter	Coefficient	Standard Error	HR	95% confidence	*p* value
				Interval for HR	
Lymph node metastasis (Negative *vs.* Positive)	0.986	0.274	2.681	1.566-4.588	<0.001
Metastasis (no *vs.* yes)	1.961	0.670	7.107	1.911-26.431	<0.01
TMEM100 level (low *vs.* high)	-0.848	0.286	0.428	0.245-0.750	<0.01

^*^The freedom of “AJCC stage” was not computable because “AJCC stage” was linear correlated with “Metastasis”.

### TMEM100 Inhibits Proliferation by Arresting G_1_/S Transition Phase of Cell Cycle and Promotes Apoptosis in CRC

**Figure 2:**
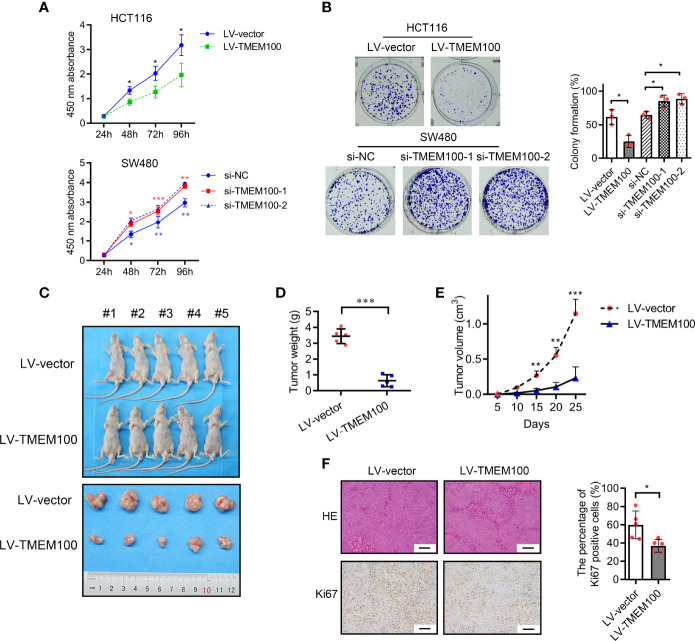
TMEM100 inhibits proliferation of CRC cells *in vitro* and *in vivo*. **(A)** CCK-8 assays were used to detect the impact of TMEM100 on the proliferation rate of CRC cells. Two-sided t-test. Red asterisks represent si-NC *vs.* si-TMEM100-1. Blue asterisks represent si-NC *vs.* si-TMEM100-2. **(B)** Colony formation assay was used to detect the impact of TMEM100 on the colony formation capacity of CRC cells. Two-sided t-test. **(C)** General observation of subcutaneous tumors in nude mice (n = 5/group) were displayed. **(D)** The weight of subcutaneous tumors in nude mice (n = 5/group) was analyzed. Two-sided t-test. **(E)** The growth curve of the subcutaneous tumors (n=5/group). Two-sided t-test. **(F)** The representative pictures of the subcutaneous tumors stained by HE and the Ki67 expression stained by IHC (n=5/group). Scale bar = 100 μm. Two-sided t-test. All data are presented as the mean ± SD. *p<0.05, **p<0.01, *** *p*<0.001.

Malignant proliferation is one of the distinct features of CRC, and we investigated whether TMEM100 impedes CRC cell growth. In this study, HCT116 cells in the TMEM100 overexpression group exhibited a lower proliferation rate than those in the control group, whereas downregulating TMEM100 in SW480 cells led to increased cell proliferation rates (**Figure 2A**). In the colony formation assays, HCT116 cells in the TMEM100 overexpression group had a diminished ability for colony formation, relative to the control group (**Figure 2B**). The downregulation of TMEM100 enhanced the colony formation capacity of SW480 cells, relative to the control group (**Figure 2B**). In the xenograft mouse models, tumors formed by the TMEM100-overexpressing HCT116 cells were found to be slow-growing and distinctly smaller than those formed by the control cells (**Figures 2C, D, E**). The IHC staining revealed that the percentage of Ki67 positive cells of subcutaneous xenograft tumors were significantly lower in the TMEM100 overexpression group, relative to control group (**Figure 2F**). These findings imply that upregulation of TMEM100 inhibits CRC cell proliferations.

**Figure 3:**
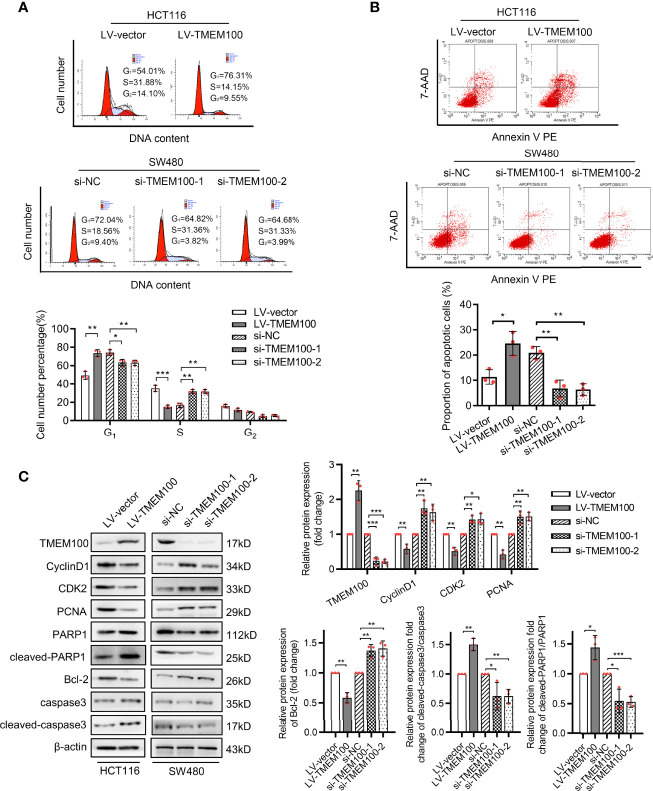
TMEM100 arresting cell cycle and potentiating apoptosis of CRC cells. **(A)** The representative pictures of the distribution of different cell cycle phases in indicated cells by flow cytometry. The percentage of cells in different phase of cell cycle was analyzed. Two-sided t-test. **(B)** The representative pictures of the percentage of cells undergoing apoptosis by flow cytometry in indicated cells. Two-sided t-test. **(C)** Western blotting analysis of TMEM100, CycilnD1, CDK2, PCNA, PARP1, cleaved-PARP1, Bcl-2, caspase3 and cleaved-caspase3 expression in indicated cells. β-actin was used as a reference control. Two-sided t-test. Data are presented as the mean ± SD. *p<0.05, **p<0.01, *** *p*<0.001.

We sought to identify the pathomechanisms through which TMEM100 inhibits CRC cell proliferation. A cell cycle analysis was conducted to determine if upregulation of TMEM100 induces the suppression of CRC cell growth by arresting a specific phase of the cell cycle. Flow cytometry revealed that HCT116 cells in the TMEM100 overexpression group exhibited a markedly low abundance of cells in S phase, relative to the control group, and the percentage of cells in G_1_ phase was markedly high (**Figure 3A**). Downregulating TMEM100 in SW480 cells enhanced the abundance of cells in S phase (**Figure 3A**). Thus, upregulation of TMEM100 seemed to inhibit G_1_/S transition during cell cycle progression. To further clarify how TMEM100 suppresses G_1_/S phase transition, key regulators related to G_1_ phase were evaluated at protein levels. It was found that upregulation of TMEM100 markedly reduced the levels of cyclin D1, CDK2 as well as PCNA proteins in HCT116 cells; however, downregulation of TMEM100 evidently increased the level of these three proteins in SW480 cells (**Figure 3C**).

Meanwhile, the results of flow cytometry analysis showed that the percentage of apoptotic cells was dramatically increased in TMEM100 overexpressing HCT116 cells, but reduced in TMEM100 downregulated SW480 cells compared with control cells (**Figure 3B**). Cleaved-PARP1 and cleaved-caspase3 are the protein markers of apoptosis process, and Bcl-2 is a distinguished anti-apoptotic protein. The results of western blotting exhibited the protein level of PARP1, cleaved-PARP1, caspase3, cleaved-caspase3 and Bcl-2 (**Figure 3C**), which indicated that cell apoptosis was promoted by TMEM100 in CRC cells. These findings imply that TMEM100 inhibits cell proliferation *via* the arrest of G_1_/S phase transitions and induces apoptosis in CRC.

### TMEM100 Inhibits the Migratory Ability of CRC Cells

**Figure 4:**
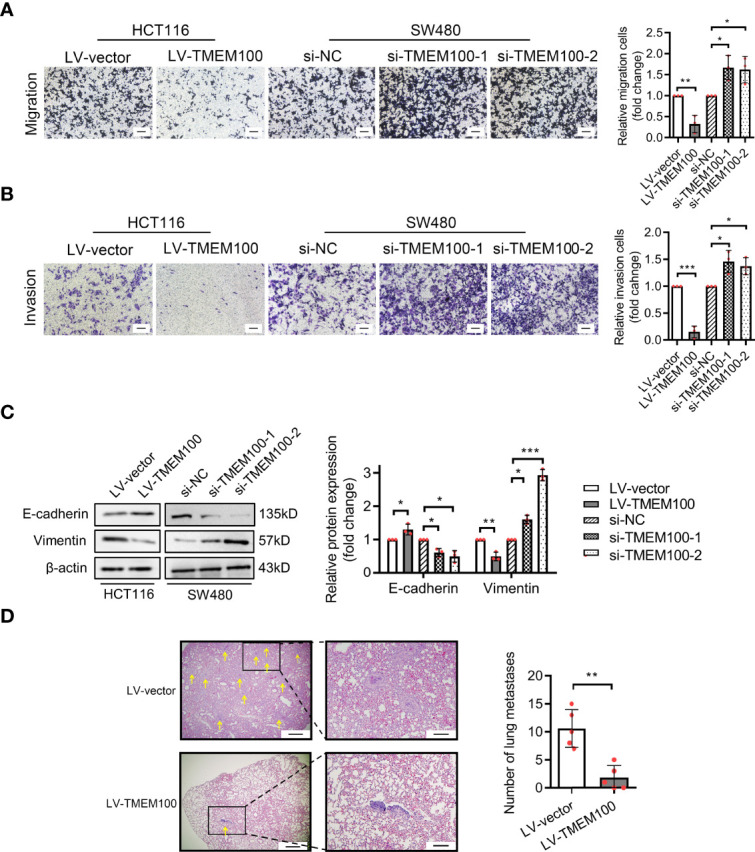
TMEM100 restrains the migration and invasion capacities of CRC cells. **(A, B)** Transwell assays were performed to evaluate the capacities of migration and invasion in indicated cells. Scale bar = 100 μm. Two-sided t-test. **(C)** Western blotting analysis of E-cadherin and Vimentin expression in indicated cells. β-actin was used as a reference control. Two-sided t-test. **(D)** The representative pictures of the metastatic neoplasm in the lung of the mice metastasis model (n=5/group). The section which presents the largest number of CRC cells colonized foci was selected, and the yellow arrow represents the foci of which diameter was a minimum of 1.5 μm. Bar in the left picture represents 5 μm, and bar in the right picture represents 1.43 μm. Two-sided t-test. Data are presented as the mean ± SD. *p<0.05, **p<0.01, ***p<0.001.

Enhanced migration is another feature of CRC. Cell migration assay revealed that migrated and invasive HCT116 cells were markedly low in TMEM100 overexpression group, relative to control group (**Figures 4A, B**). For SW480 cells, the numbers of migrated and invasive cells were significantly high in si-TMEM100 groups, relative to si-NC groups (**Figures 4A, B**). Vimentin and E-cadherin are epithelial to mesenchymal transition (EMT) biomarkers, and western blotting analysis revealed that E-cadherin levels in HCT116 cells were elevated and Vimentin levels were low in TMEM100 overexpression group, compared to control group (**Figure 4C**). Consistently, E-cadherin as well as vimentin levels exhibited opposite patterns when TMEM100 was downregulated in SW480 cells (**Figure 4C**). Then, we evaluated the *in vivo* effects of TMEM100 on migratory capacities of CRC cells by injecting mice tail veins with HCT116 cells infected with LV-vector or LV-TMEM100 lentivirus. Histology showed that the abundance of metastatic tumors in lungs of TMEM100 overexpression group were low, relative to control group (**Figure 4D**). These findings imply that TMEM100 suppresses metastasis of CRC cells by regulating EMT.

### TMEM100 Is Negatively Associated With HIF-1α Protein Levels in CRC Tissues

**Figure 5:**
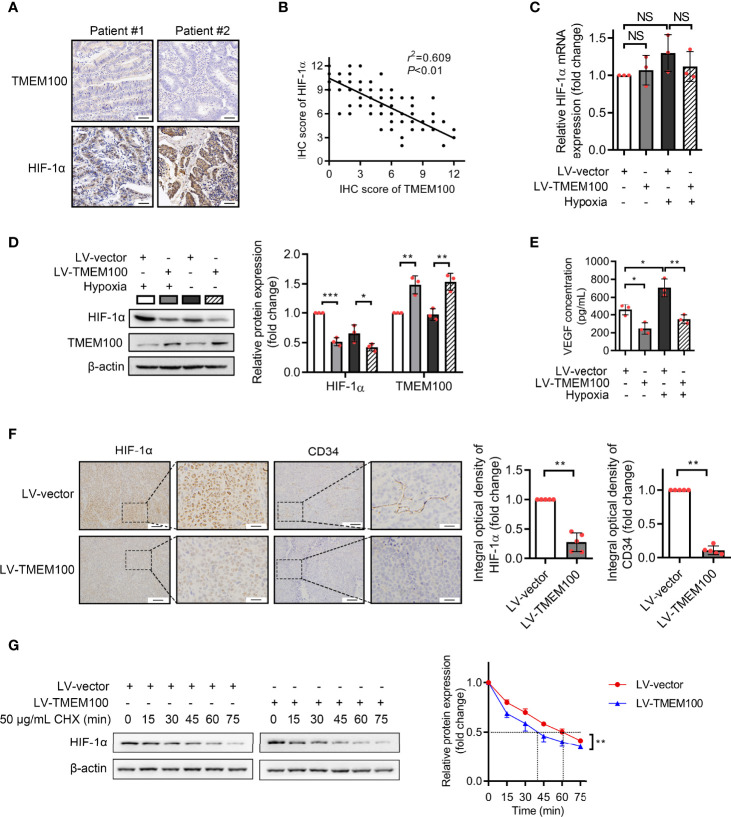
TMEM100 overexpression leads to a reduction in HIF-1α content and microvascular density in CRC. **(A)** Representative images of IHC staining of TMEM100 and HIF-1α in CRC tissues. Scale bar = 100 μm. **(B)** The correlation between TMEM100 and HIF-1α expression in CRC specimens was analyzed, and the linear correlation coefficient and statistical significance were indicated. **(C)** HCT116 cells were infected with LV-vector or LV-TMEM100 lentiviruses. The relative mRNA expression of HIF-1α was detected by RT-qPCR in indicated cell under normoxic and hypoxic conditions. ANOVA. **(D)** TMEM100 and HIF-1α expression in indicated cell was detected by western blotting. β-Actin was used as a reference control. Two-sided t-test. **(E)** The extracellular concentration of VEGF released by indicated cells was detected by ELISA. ANOVA. **(F)** The representative pictures of the HIF-1α and CD34 expression of subcutaneous tumors (n=5/group) stained by IHC. Two-sided t-test. **(G)** Control or TMEM100 overexpression cells were incubated with cycloheximide (CHX, 50 μg/mL) for the indicated time. The cell lysates were then analyzed by western blotting. The dot lines indicated the half-life of the HIF-1α. β-actin was used as a reference control. Two-sided t-test. Data are presented as the mean ± SD. *p<0.05, **p<0.01, ***p<0.001. NS, no significance.

We presented the potential association of TMEM100 with HIF-1α in the introduction. The excessive accumulation of HIF-1α further exerts its cancer-promoting effect under hypoxia. HIF-1α accumulation can be induced when cells were cultured under 1% O_2_ condition for 12 h *in vitro* (13, 19). To establish the significance of TMEM100 in CRC, we evaluated HIF-1α levels in a tissue microarray with the same catalog number as that used to evaluate TMEM100 levels *via* IHC. TMEM100 protein levels were negatively associated with HIF-1α levels (**Figures 5A, B**). The mRNA levels of HIF-1α showed no changes when TMEM100 was overexpressed (**Figure 5C**). However, HIF-1α protein levels were markedly low under normoxic and hypoxic environments in the TMEM100 overexpression group, relative to the control group (**Figure 5D**). Vascular endothelial growth factor (VEGF) is among the HIF-1α target genes, and VEGF secreted by cells binds to its receptor and induces the generation of new blood microvessels. Overexpressions of TMEM100 markedly reduced VEGF release in HCT116 cells under normoxic and hypoxic conditions (**Figure 5E**). We then examined the influence of TMEM100 on HIF-1α protein content and microvascular density in subcutaneous xenograft tumors. The detection of endothelial cell markers on microvessels is one of the recognized methods to evaluate tumor angiogenesis. For example, CD34 has been used to evaluate angiogenesis in many malignancies. The IHC staining revealed that HIF-1α protein levels of subcutaneous xenograft tumors were significantly lower, and the microvascular density marked by CD34 was drastically suppressed in the TMEM100 overexpression group, relative to control group (**Figure 5F**). Therefore, we confirmed that the overexpression of TMEM100 led to a reduction in HIF-1α content and microvascular density.

### TMEM100 Inhibits the Migration and the Angiogenesis Induction Capacities of CRC Cells by Enhancing HIF-1α Degradation *via* Ubiquitination/Proteasome Pathway

**Figure 6:**
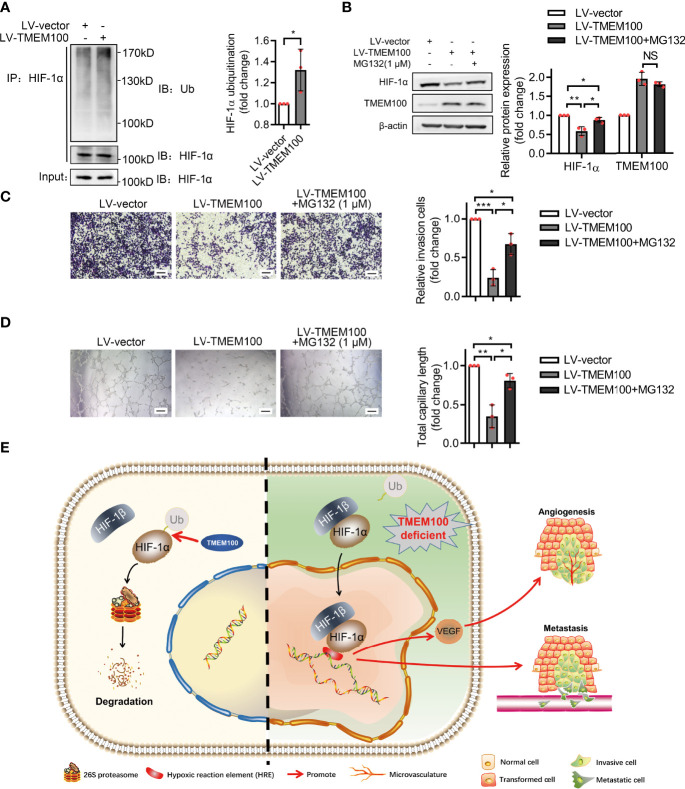
TMEM100 inhibits the migration and angiogenesis induction capacities of CRC cells by promoting the degradation of HIF-1α *via* ubiquitination/proteasome pathway. **(A)** HCT116 cells were infected with LV-TMEM100 or LV-vector lentiviruses, and then cultured for 48 h under hypoxic conditions. Cell extracts were immunoprecipitated (IP) using anti-HIF-1α antibody and blotted with anti-ubiquitin antibody. HIF-1α was used as a reference control. Two-sided t-test. **(B)** All cells were grown in hypoxic environments for a total of 48 h. For cells in “LV-TMEM100 + MG132” group, MG132 (1 μM) was supplemented to the medium. The cell lysates were then analyzed by western blotting. β-actin was used as a reference control. Two-sided t-test. **(C)** Transwell assays were performed to evaluate the migration capacities of cells in indicated groups under hypoxic conditions. Scale bar = 100 μm. Two-sided t-test. **(D)** The *in vitro* tube formation assays were performed to evaluate the angiogenesis induction capacity of HCT116 cells in indicated groups. Two-sided t-test. **(E)** The schematic representation of TMEM100 promoting HIF-1α degradation *via* ubiquitination/proteasome pathway. HRE means hypoxia-response element. Data are presented as the mean ± SD. *p<0.05, **p<0.01, ***p<0.001. NS, no significance.

Given that TMEM100 had no influence on the mRNA level of HIF-1α during normoxia and hypoxia, which indicated that TMEM100 had no effect on HIF-1α transcription, we assessed whether TMEM100 impacts HIF-1α degradation. Cycloheximide (CHX), a common reagent used to inhibit protein synthesis, blocks the elongation phase of eukaryotic translation. To further reveal whether TMEM100 regulated HIF-1α stability, we treated CRC cells with CHX to prevent protein synthesis. Overexpressed TMEM100 shortened the half-life of HIF-1α from 61 min to 40 min, which hinted that TMEM100 might be involved in HIF-1α degradation processes (**Figure 5G**). The ubiquitin–proteasome degradation system is the major pathway of HIF-1α degradation. HIF-1α was immunoprecipitated from cells, and the abundance of HIF-1α-linked ubiquitin evaluated by immunoblotting with an anti-ubiquitin antibody. The amounts of HIF-1α-conjugated ubiquitin from TMEM100-overexpressing cells were markedly low, compared with control cells during hypoxia (**Figure 6A**). We further tested whether TMEM100 inhibits HIF-1α degradation *via* the proteasome. TMEM100-overexpressing HCT116 cells were treated with MG132, an inhibitor of proteasome, under hypoxia. The results showed that overexpressing TMEM100 significantly decreased HIF-1α levels. However, in the presence of MG132, TMEM100 overexpression did not suppress HIF-1α levels, implying that TMEM100 inhibits HIF-1α accumulation in a proteasome-dependent manner (**Figure 6B**). Thus, TMEM100 promotes HIF-1α degradation *via* the ubiquitination–proteasome pathway in CRC cells.

We next verified the relationship of TMEM100, HIF-1α, angiogenesis and migration in CRC. HCT116 cells were grown in hypoxic conditions, and the TMEM100 overexpression group showed fewer migrating cells than the control group (**Figure 6C**). Then cells were treated with MG132 to block the ubiquitin/proteasome degradation of HIF-1α. Cells in the “TMEM100+MG132” group showed significant more migrating cells than those in the TMEM100 overexpression group, but considerable migrating cells relative to the control group (**Figure 6C**). *In vitro* tube formation assay revealed that cells in TMEM100 overexpression group showed weaker angiogenesis induction capacity, relative to the control group (**Figure 6D**). Cells in the “TMEM100+MG132” group showed significantly enhanced angiogenesis induction capacity compared with the TMEM100 overexpression group (**Figure 6D**). Thus, overexpressed TMEM100 suppressed the migration as well as angiogenesis induction capacity of CRC cells, but these effects were reversed by blocking the ubiquitin/proteasome degradation pathway process using MG132, which hinted that TMEM100 inhibited the migration and the angiogenesis induction capacities of CRC cells by impacting HIF-1α degradation *via* the ubiquitination/proteasome pathway (**Figure 6E**).

## Discussion

CRC has a relatively diverse genetic background and pathological characteristics. The currently recognized CRC pathogenesis mainly involves the chromosomal instability, microsatellite instability and serrated pathways (20). These pathways ultimately lead to the imbalance of proliferation and apoptosis and the gain of invasion and migration properties in cancer cells (21–23). *APC* is mutated in most CRC, and HIF-1α directly suppresses the *APC* promoter by occupying a specific site, causing decreased mRNA and protein levels of APC (15). Meanwhile APC reduces HIF-1α mRNA in a β-catenin l-dependent manner, implying that HIF-1α downregulates APC further improves tumor cell survival under hypoxia (24). And the functional loss of APC caused by APC mutation facilitates cancer cell survival by inducing HIF-1α expresses. The effect of TMEM100 in inhibiting HIF-1α accumulation interfered above vicious circle, and impeded the progression of CRC ultimately. CRC cells are commonly characterized by uncontrolled proliferation, invasion, and metastasis (25). Increased proliferation caused by oncogenic mutations is mediated by genetic as well as epigenetic alterations in the apoptotic pathway, ultimately leading to uncontrolled tumor growth, as reflected in shortened cell mitotic time in terms of the cell cycle. TMEM100, in contrast, caused CRC cell cycle arrest. The main effect caused by rapid proliferation of tumor tissues can affect intestinal peristalsis, digestion and absorption, and even cause intestinal obstruction. Moreover, due to the loss of body nutrients caused by the rapid growth of tumor tissues, patients often show cachexia in the later stage of CRC. Invading or metastatic cancer cells grow in other tissues or organs, such as common liver and lung metastases, which more seriously prevent other tissues or organs from exercising their normal function. Metastatic cancer also brings even more severe challenges to the treatment of cancer. At the molecular biology level, migration and invasion are other prominent features of cancer cells including CRC, and EMT leads to a tendency towards metastasis and invasion (26, 27). However, TMEM100 inhibited the EMT process of HCC, lung cancer, PC and CRC.

Tumor proliferation and invasion are closely related to angiogenesis in cancer tissues. With the rapid proliferation of cancer cells, unfavorable factors such as low oxygen, low pH, and metabolite accumulation inside the tumor tissue cause cancer cells to actively induce the formation of neovascularization, and cancer cells that break through the neovascular basement membrane can be transferred to other tissues or organs *via* the bloodstream (28). Newly formed blood vessels are more conducive to the metastasis of cancer cells, as the neovascular base membrane is thinner and easier to penetrate, thus allowing cancer cells to enter the blood circulation and spread to other tissues, while relatively mature vascular wall cancer cells are difficult to penetrate. It has been established that a higher density of blood vessels in tumors increases the possibility of vascular infiltration in cancer cells and favors cancer cell spread and distant metastasis (29).

Angiogenesis is induced by HIF-1α through the activation of target genes, including VEGF in CRC. Under insufficient oxygen supply conditions, HIF-1 activates the expressions of VEGF genes involved in migration and colonization of vascular endothelial cells (30). Activation of VEGF allows for the continuity of neovascularization in CRC tissue with the host vasculature (31). We established that TMEM100 was inversely associated with protein levels of HIF-1α in CRC tissues, further indicating that TMEM100 promotes the ubiquitin/proteasome degradation pathway of HIF-1α and reduces the VEGF release, a downstream gene of HIF-1α, which subsequently inhibits CRC cell migration and induced angiogenesis. CRC cells achieve survival in different hypoxic states through the VEGF/KDR/HIF-1α autocrine circuit (32). The reduction in HIF-1α content and VEGF release in CRC cells caused by TMEM100 disrupted this circuit and decreased the microvessel density. Microvessel density has been shown to be an important factor in patient prognosis in a variety of malignancies including CRC (28, 33). TMEM100 reduced the microvessel density in the subcutaneous graft tumors.

Molecular targeted drugs are newly emerging therapeutics for CRC (34), and one of the antitumor effects of cetuximab is inhibition of the PI3K pathway which in turn downregulates HIF-1α synthesis and activity (35). In emerging molecular treatment regimens, the use of biological agents including VEGF inhibitors and EGFR inhibitors is gradually showing strong therapeutic effects. Therefore, the inhibition of HIF-1α accumulation and VEGF release by TMEM100 suggests that TMEM100 is a potential treatment target for CRC. A study revealed that TMEM100 modulates TGF-β signaling pathway in CRC. Our proposal that TMEM100 participates in the CRC angiogenesis process by affecting the degradation of HIF-1α is a completely new research direction.

In addition, TMEM100 is an important downstream gene of the BMP9/BMP10 signaling pathway during the establishment of primitive cardiovascular system. In oncology, BMP signal acts as a tumor suppressor in intestinal adenoma formation, and the inhibition of BMPs promotes EMT (36). Knockdown of BMP receptors in epithelial or stromal cells leads to excessive proliferation of intestinal epithelial cells and the formation of precancerous polyps (36, 37). And TMEM100 also showed the function of inhibiting the migration and EMT process in CRC. The relationship between BMPs and TMEM100 in CRC is intriguing.

## Data Availability Statement

The original contributions presented in the study are included in the article/supplementary material. Further inquiries can be directed to the corresponding authors.

## Ethics Statement

The studies involving human participants were reviewed and approved by the Clinical Research Ethics Committee of the Second Affiliated Hospital of Xi’an Jiaotong University. The patients/participants provided their written informed consent to participate in this study. The animal study was reviewed and approved by Animal Care Committee of Xi’an Jiao Tong University.

## Author Contributions

YZ designed the experiments, supervised the study, revised the paper and provided the material support. YZ and YTZ completed the main experiments and wrote the first draft of the paper. JJ prepared the figures and analyzed the data. LD and BZ helped with the preparation of human samples. All authors have reviewed the final version of the manuscript and approve it for publication.

## Funding

The present study was supported by the Grants from Shaanxi Provincial Central Committee’s Special Fund Plan for Guiding Local Science and Technology Development”, No. 2016ZY-HM-01.

## Conflict of Interest

The authors declare that the research was conducted in the absence of any commercial or financial relationships that could be construed as a potential conflict of interest.

## Publisher’s Note

All claims expressed in this article are solely those of the authors and do not necessarily represent those of their affiliated organizations, or those of the publisher, the editors and the reviewers. Any product that may be evaluated in this article, or claim that may be made by its manufacturer, is not guaranteed or endorsed by the publisher.
